# Recent Advances in Functional Materials for Optical Data Storage

**DOI:** 10.3390/molecules29010254

**Published:** 2024-01-03

**Authors:** Dihua Dai, Yong Zhang, Siwen Yang, Weicheng Kong, Jie Yang, Jijun Zhang

**Affiliations:** 1 China Hualu Group Co., Ltd., 717 Huangpu Road, Dalian 116023, China; daidh@hualu.com.cn (D.D.); zhangy16@hualu.com.cn (Y.Z.); yangsw@hualu.com.cn (S.Y.); kongwch@hualu.com.cn (W.K.); 2 School of Life Sciences, Jilin University, 2699 Qianjin Street, Changchun 130012, China

**Keywords:** optical data storage, functional materials, nanoparticles, graphene, diarylethene

## Abstract

In the current data age, the fundamental research related to optical applications has been rapidly developed. Countless new-born materials equipped with distinct optical properties have been widely explored, exhibiting tremendous values in practical applications. The optical data storage technique is one of the most significant topics of the optical applications, which is considered as the prominent solution for conquering the challenge of the explosive increase in mass data, to achieve the long-life, low-energy, and super high-capacity data storage. On this basis, our review outlines the representative reports for mainly introducing the functional systems based on the newly established materials applied in the optical storage field. According to the material categories, the representative functional systems are divided into rare-earth doped nanoparticles, graphene, and diarylethene. In terms of the difference of structural features and delicate properties among the three materials, the application in optical storage is comprehensively illustrated in the review. Meanwhile, the potential opportunities and critical challenges of optical storage are also discussed in detail.

## 1. Introduction

The unavailable long-life, low-energy, super high-capacity, and renewable and sustainable optical data storage remains a severe challenge to be conquered, which promotes researchers to spare no efforts in designing and fabricating novel systems using more remarkable optical storage materials [1,2,3,4,5]. So far, the total amount of annual data produced globally has increased very fast, which has doubled every two years since 2000 [6]. The traditional technologies including magnetic storage and electrical storage have been improved to deal with the explosive growth of mass information; however, the commercialized products are mainly developed via the two technologies with limited capacity, and the strategies for expanding capacity have reached a bottleneck that is extremely hard to be broken [7,8]. Moreover, the serious drawbacks such as strict storage conditions, the high energy consumption of equipment, and low-security level have restricted the further improvement of magnetic storage and electrical storage, which are difficult to adapt to the booming information era [9,10]. Optical storage materials have been one of the most common recording mediums since the beginning of the 21st century, accompanied by the rapid development of laser technology. Optical storage is the technology that is based on the interaction between laser and recording medium, and the investigation on breaking the diffraction limit for conquering the challenge of present data storage has attracted extensive attention in information technology industry [11]. Compared to the traditional means, optical storage technology shows more possibility for satisfying the requirements of data storage equipped with the properties including large capacity, high safety, intense stability, reasonable price, and low energy consumption [12]. In the past decade, researchers have devoted themselves to exploring new functional materials to be applied in recording media. Meanwhile, the representative recording media such as rare-earth doped upconversion nanoparticles (UCNPs) [13,14,15,16,17], graphene derivatives (GDs) [18,19,20,21], and diarylethene derivatives (DTDs) [22,23,24,25,26] are the most potential materials to be further investigated, which are promising to facilitate the development of optical storage technology and exploit valuable strategies for practical applications and industrialized projects [27,28,29,30,31,32].

The low-energy near-infrared light can be transferred to high-energy UV light or visible light by using the functionalized UCNPs, which is developed for the applications in photolithography, photothermal therapy, photoswitch, and optical storage [33,34]. The UCNPs possess distinct fluorescence properties, which can be incorporated into luminescent materials such as quantum dots, organic dyes, and aggregation-induced emission luminogens (AIEgens) to fabricate novel recording materials equipped with large memory capacities. In comparison with common organic fluorescence chromophores, the UCNPs possess wider energy levels to further reduce transition rates, enabling the low power-assisted stimulated emission depletion (STED) effects to play a crucial role in optical storage [35].

So far, the standardization production of graphene has received remarkable achievements, and the representative industrial process derived from chemical vapor deposition and epitaxial growth has gained rapid development [36,37,38]. In order to overcome the challenge of production modes, the architecture of graphene-based materials like graphene nanobelt and graphene quantum dots has been widely reported to be applied in the construction of field effect transistors, bioimaging, and optical writing [39]. Thus, the significance of designing new graphene nanostructures is in urgent need to cooperate with super-resolution microscopy technology, for the extensive imaging of GD substrates. The majority of receptor systems only can be quenched in a narrowed spectral range; however, the GDs are equipped with the feature of broadband absorption to exhibit energy transfer in the whole visible spectrum, possessing great potential to be introduced in macromolecular systems to prepare composite materials for the application in optical storage [40].

Moreover, on the basis of the established UCNPs, the inorganic–organic hybrid materials are well constructed, combining the merits of UCNPs and organic stimuli-responsive molecules for proposing a new approach to advanced optical storage [41]. The DTDs are the most used organic molecules for optical writing because of their photo-isomerization properties. As the typical photoresponsive molecules, DTDs show vital values in the practical application of the reversible memory assisted by the photoswitched “writing–reading–erasing” [42]. Generally, DTDs can finish the rapid transformation between open and close conformations irradiated by the UV light and visible light, respectively. More importantly, DTDs have favorable physicochemical properties, including strong thermal stability, moderate fatigue resistance, a quick responsiveness and reaction rate, a high-conversion ratio of open/close isomers, sufficient quantum yield, and an obvious difference of absorption wavelength between the open/close isomers. The DTD-based composites play a crucial part in optical storage owing to their significant changes of absorption spectra, dielectric constants, and geometrical configuration, and have tremendous commercial values for optical storage revolution in the future [43,44,45].

The optical storage materials are one of the most promising recording media in the digital age [46]. Researchers have been sparing no efforts on the in-depth exploration of the three functional memory materials for pursuing a larger storage density [47,48,49,50,51]. According to the strategies of increasing the number of layers, enhancing the recording dimensions of recording media, and narrowing the diffraction limit, we envision that the ultrahigh storage density of the TB level, even PB level, will eventually be approached in a single disc [6,52].

**Scheme 1:**
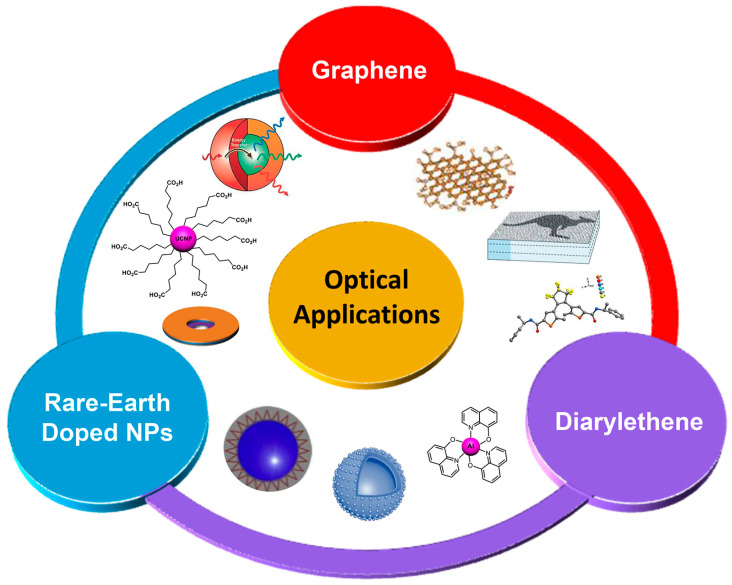
The three functional materials containing UCNPs, GDs, and DTDs for applications in optical data storage.

The volume of global data generated from the internet and portable devices will reach 175 zettabytes (10^9^ TB) estimated by the International Data Corporation, and the massive information will need to be continuously stored and facilely accessed over long-term periods [6]. Thus, the age of data explosion requires the researchers to lay great emphasis on the functional materials that will hopefully be used for practical application and even in commercial production within a decade or two, for satisfying the urgent need of increasing the capacity of currently available optical devices. Considering the attractive strategies of enlarging storage capacity, in this review, we outline three categories of materials including UCNPs, GDs, and DTDs for elaborately discussing their optical properties and interaction mechanisms, and mainly highlighting their application in optical data storage (Scheme 1). Meanwhile, the perspectives, advantages, and disadvantages of the three materials are also addressed. We hope that this review will be helpful for researchers to stimulate new thinking for designing novel platforms, to expand the optical storage capacity, open an avenue for synthesizing valuable materials in practical use, and understand the future development of the research field of optical storage.

## 2. UCNP-Based Functional Materials

The technology of high-density optical writing is of great significance in data storage. Additionally, the optical writing technology at nanoscale level based on the far-field super-resolution method provides a unique approach for dealing with memory devices with a large capacity. However, the current nanoscaled optical writing measures generally rely on the mechanism of photo-initiation and photo-inhibition, which seriously restricts their further development due to the disadvantages of the high intensity of laser, large consumption of energy, and short life of devices [53]. Notably, the UCNP-based systems have broad excitation levels to decrease transition rates. Meanwhile, according to the far-field super-resolution technology, the electron transition in UCNP-based systems can be selectively modulated to activate the energy transfer-derived low-power radiation for breaking through the diffraction limit in optical writing. In this section, the diverse UCNP materials used for optical writing and optical storage with particular functions are discussed in detail (Table 1).

**Figure 1:**
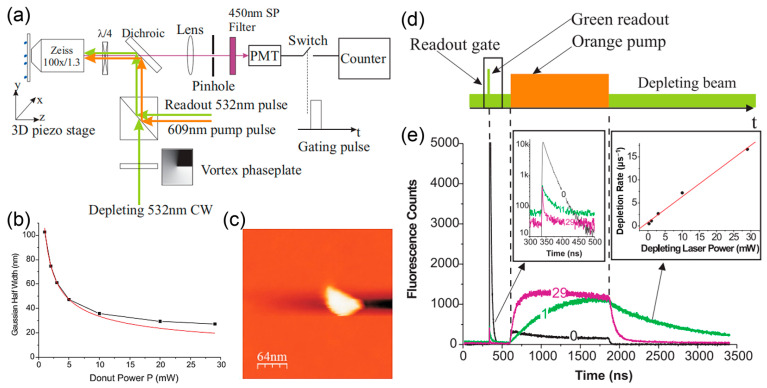
(**a**) The confocal experiments and the time-domain studies of UCNPs-1; (**b**) the correlation of the Gaussian half width of high-resolution fluorescence feature; (**c**) morphology characterization of UCNPs-1; (**d**) the examination of the efficiency of intermediate state depletion of UCNPs-1; (**e**) fluorescence signals of UCNPs-1 corresponding to different light intensities [54].

Kolesov and coworkers prepared a background-free subdiffraction optical microscopy and Pr^3+^-doped yttrium aluminium garnet (YAG)-based UCNPs in 2011 (Figure 1a) [54]. Subsequently, employing the microscopy technology of Pr:YAG nanoparticles (UCNPs-1), the UCNPs-1 was capable of emitting upconversion UV radiation for achieving the background-free microscopy, and utilized the donut-shaped beam to obtain the subdiffraction-limited optical resolution. Owing to the restriction of the particle size of UCNPs-1, all the optical resolution achieved 50 nm (Figure 1b,c). Moreover, the new-designed technology using UCNPs-1 possessed great photostability, and the technology was similar to the STED microscopy. However, the working mechanism of the as-prepared technology was explained as stimulated absorption instead of stimulated emission, which was different from the STED technology. Furthermore, the UCNPs-1 comprising the YAG as building blocks had no toxic side effects on cells, which was a promising candidate for background-free cell imaging at the nanoscale (Figure 1d,e). In comparison with the UV spectra to kill cells, the UV irradiation emitted from Pr^3+^ had a large red shift, so the low dose of Pr^3+^ could be recorded as a nontoxic substance. Thus, through the upconversion imaging strategy of intermediate electronic state depletion at the nanoscale, more promising photostable rare-earth doped upconversion labels will be fabricated with an emission wavelength in the range of visible light.

**Figure 2:**
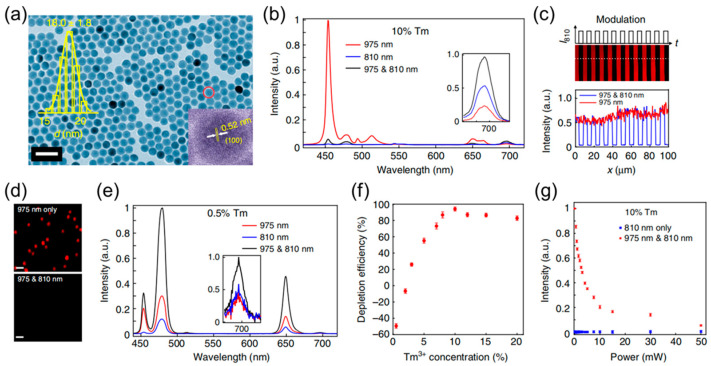
(**a**) Transmission electron microscopy (TEM) images of UCNPs-2; (**b**) depletion efficiency of UCNPs-2; (**c**) the upconversion imaging of UCNPs-2 film with laser co-irradiation; (**d**) the UCNPs-2 image with/without 810 nm laser co-irradiation; (**e**) the fluorescence spectra of UCNPs-2 under the different excitation wavelength; (**f**) the depletion efficiency of UCNPs-2 with different Tm^3+^-doped concentrations; (**g**) the curve of depletion efficiency of UCNPs-2 [55].

The optical depletion of upconversion emission has been a great challenge in the field of material science, which is attributed to a depletion laser wavelength matching well with multiple energy gaps, complicating the emission intensity and depletion efficiency of depletion laser. In terms of resolving the problem, He, Zhan, and coworkers discovered unique lanthanide-doped UCNPs, which were capable of efficient emission depletion by ion-assisted cross relaxation, dramatically reducing the requirement of optical depletion for laser intensity (Figure 2a) [55]. The as-prepared UCNPs employed the ion–ion interactions to prohibit depletion laser enhancement and improve the depletion effects. The NaYF_4_:18% Yb^3+^, 10% Tm^3+^ nanoparticles (UCNPs-2) successfully achieved 96% of the depletion efficiency at 455 nm with blue emission under 810 nm irradiation. The intense cross relaxation in low-lying states of emitters, from ^3^H_4_ + ^3^H_6_ to ^3^F_4_ + ^3^F_4_, played an important role in the optical inhibition of 455 nm emission (Figure 2b,c). Through management of Tm^3+^ emission assigned to UCNPs-2 applied in other channels, the utilization of a single pair of excitation/depletion laser beams could realize two-color super-resolution imaging (Figure 2d). Moreover, the UCNPs-2-based upconversion STED fluorescence microscope distinctively lowered the requirement of depletion power density and eliminated the demand on pulsed laser, which endowed the STED fluorescence microscope with higher biocompatibility, and provided the alternative approach for conventional methods that used organic dyes and other STED imaging measures based on luminescent nanoparticles. Thus, this work not only stimulates new thinking for exploring the optical modulation of luminogens to be further applied in super-resolution imaging, but possesses great potential for expanding the applied range of UCNPs in fields of optical storage, nanoscale photonics, and life science.

**Figure 3:**
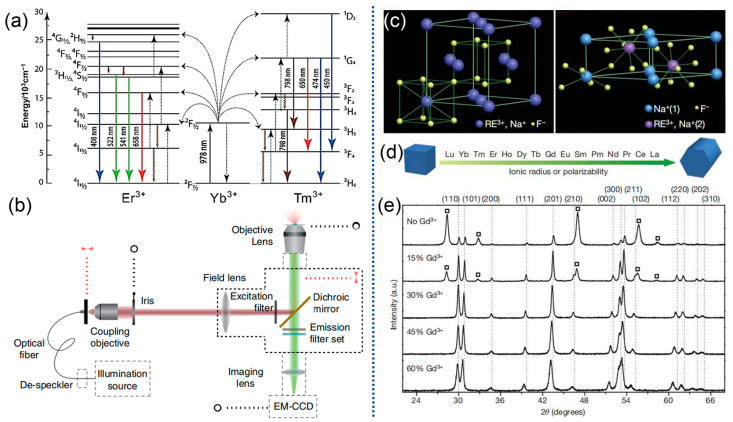
(**a**) Schematic diagram of energy level of Yb^3+^ with Er^3+^ or Tm^3+^ in UCNPs-3; (**b**) schematic representation of the improved optical microscopy and optical circuits [56]; (**c**) simulative structures of cubic- and hexagonal-phase NaYF_4_; (**d**) transition from cubic phase to hexagonal phase of nanocrystals; (**e**) XRD patterns of the NaYF_4_:Yb/Er nanocrystals with various concentration of Gd^3+^ [57].

Song and coworkers designed and synthesized luminescent nanomaterials of NaYF_4_:Yb,Er and NaYF_4_:Yb,Tm (UCNPs-3) for optical imaging in skin [56]. The energy transfer upconversion exhibited that Yb^3+^ activated the radiation at 978 nm and Tm^3+^ radiated at 474 nm and 798 nm spectral bands (Figure 3a). Owing to the back scattering of excitation emission and initiative fluorescence of biological tissue, the new-designed nanomaterials could completely prohibit the background. During the experiments, the parts of freshly excised and microneedle-treated skin samples were coated with oil-generated UCNPs-3 for optical imaging. The freshly excised skin sample showed that 32 nm and 8 nm of UCNPs-3 stayed at the cuticle on top of the skin. Meanwhile, the microneedle-treated skin sample exhibited that 8 nm of UCNPs-3 was located at the diffusion-formed depression. By virtue of the wide-field luminescence optical microscopy and suppressing the background level to electronic noise, the optimal imaging contrast ratio of UCNPs-3 could be obtained, superior to the present optical labels (Figure 3b). Thus, taking advantage of the high-resolution imaging comparison between UCNPs-3 and UV organic fluorescent dye model, the as-prepared UCNPs-3 revealed remarkable superiority and extended the application of UCNPs in skin imaging and data writing, which had great significance of super-resolution imaging and high-density optical storage.

In terms of nanocrystals, the doping method poses critical importance for stabilizing the specifical phase, changing the electronic property, modulating magnetics, and regulating emission features. Liu and coworkers developed a doping system for affecting crystal growth, which could simultaneously control the phase, size, and optical emission features of as-prepared nanocrystals [57]. According to the control experiments, the introduction of trivalent lanthanide-doped ions could accurately regulate the comprised concentration (Figure 3c). Moreover, the NaYF_4_ nanocrystal could reasonably regulate its size around 10 nm, transform the crystalline phase to a cubic/hexagonal phase on demand, and guarantee the color of upconversion emission from green to blue (Figure 3d). The novel strategy was able to conveniently obtain the exact phase and size and immensely improve the controllability with smaller feature size and ideal optical property via changing the doping concentration. Overall, it was worth noting that this work provided an efficient way for green synthesis and conveniently preparing NaYF_4_ upconversion nanocrystals with an extra small hexagonal phase. Furthermore, the PXRD patterns showed that the paramagnetic Gd^3+^ dopant endowed the nanocrystals with a capability for a magnetic resonance imaging probe (Figure 3e). The doping-induced structural and size transformation of NaYF_4_ upconversion nanocrystal could be further utilized in other rare-earth doped nanocrystal systems for exploring luminescent labels and optical memory devices.

**Figure 4:**
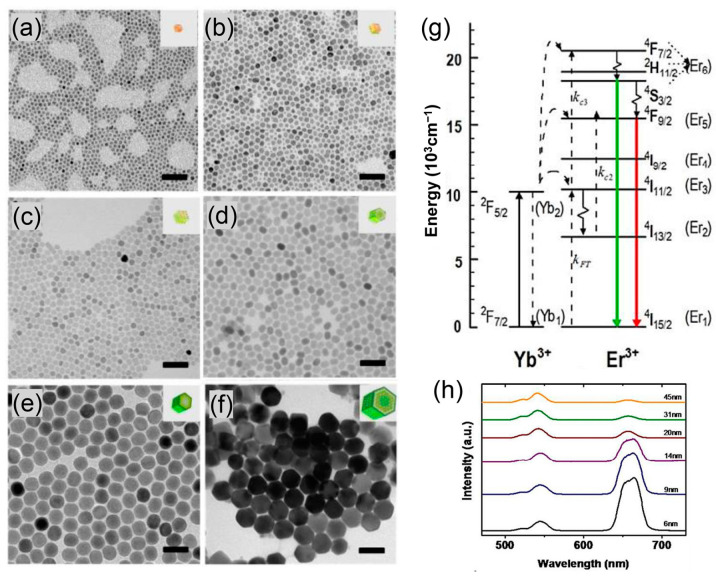
(**a**–**f**) TEM images of NaYF_4_:Yb,Er nanocrystals with different diameters; (**g**) radiative/nonradiative transitions and energy transfer processes in NaYF_4_:Yb,Er nanocrystals; (**h**) fluorescent spectra of NaYF_4_:Yb,Er nanocrystals with different sizes [58].

For another instance, Goldys, Jin, and coworkers studied on the various upconversion luminescent intensity of NaYF_4_:Yb,Er (cubic phase to hexagonal phase) with the sizes from 6 nm to 45 nm (Figure 4a–f) [58]. The experimental results indicated that with the decrease in nanocrystal size, the fluorescent lifetime subsequently reduced while the luminescent intensity of red and green emission gradually increased. Additionally, the nanocrystal emitters were distributed as a model at the near-surface and inner area of each nanocrystal, for quantifying the effect of the surface on the fluorescent lifetime. The four nonradiative recombination including intrinsic phonon modes, solvent-mediated quenching, the vibration energy of surface ligands, and surface defects were further investigated to explore the effects of the mechanisms toward decay rates of different sizes, and the results illustrated that the defect density was the main factor to direct the decay rate of rare-earth doped nanocrystals below 15 nm (Figure 4g,h). In detail, this work certified that high-quality β-particles behavior was corresponding to the three models of vibration energy of surface ligands, solvent-mediated quenching, and surface defects, while the decay time of smaller α-particles relied on the defect intensity. This work adopted defect-reduction strategies to stimulate new thinking for producing smaller and brighter upconversion nanocrystals, which possessed great potential to lower the diffraction limit in optical writing.

**Figure 5:**
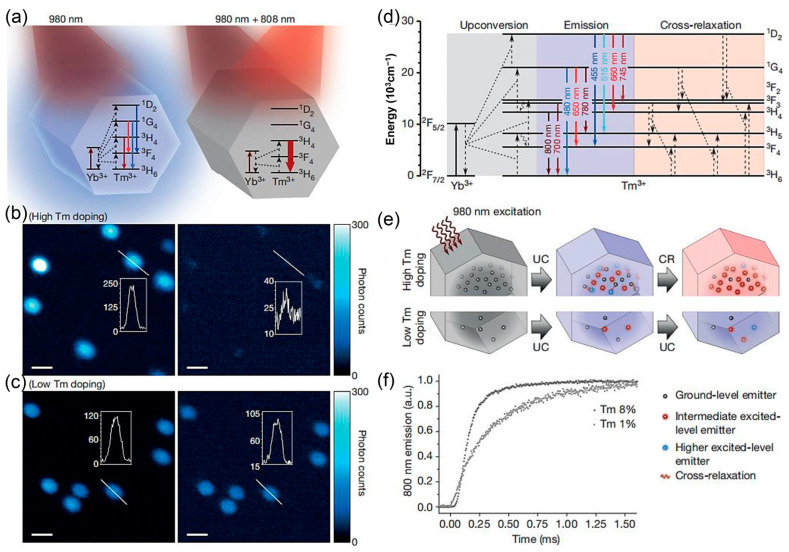
(**a**) Energy level representation of Yb/Tm doped UCNPs-4 under different excitation wavelength; upconversion emission of the (**b**) 8% Tm-doped and (**c**) 1% Tm-doped UCNPs-4; (**d**) energy level diagram of Yb/Tm doped UCNPs-4; (**e**) schematic illustration of the photon avalanche process; (**f**) transient response of the upconverted emission from Tm-doped UCNPs-4 [30].

Subsequently, Jin, Xi, Lu, and coworkers utilizing high-concentration doped rare-earth upconversion nanoparticles (UCNPs-4) successfully realized the super-resolution results of low-stimulated radiation quenched fluorescence, by virtue of photon avalanches effect triggered stimulated radiation enhancement (Figure 5a) [30]. Compared with the traditional stimulated radiation quenched super-resolution intensity, the new-designed strategy decreased the fluorescent intensity with 2~3 order of magnitudes, and achieved 28 nm super-resolution by 30 mW continuous laser. Moreover, the narrowed distance among the emitters could cause intense cross relaxation to trigger photon avalanches filling the metastable state ^3^H_4_ level, leading to the particle inversion at ^3^H_6_ original level in a single nanoparticle and fluorescence quenching (Figure 5b,c). The experimental results indicated that the corresponding upconversion band transition from the ^3^H_4_ to ^3^H_6_ level under 808 nm radiation can drive enhanced stimulated emission and release the ^3^H_4_ intermediate level, generating the inhibited blue emission during the upconversion process (Figure 5d,e). Combining the luminescent properties of UCNPs and the mechanism of the stimulated radiation quenching of the intermediate level, the 28 nm super high resolution was successfully realized on the 13 nm and 40 nm samples (Figure 5f). Thus, the high-doped UCNPs-4 and upconversion STED technology are hoped to play important parts in high-density optical data storage.

**Figure 6:**
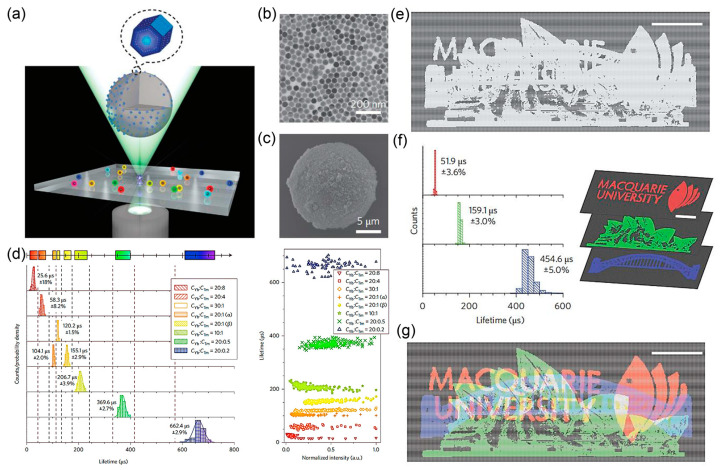
(**a**) The flow chart of Tm upconversion nanocrystals introduced into the porous microspheres; (**b**) TEM image of the nanocrystals and (**c**) scanning electron microscopy (SEM) image of incorporating the nanocrystals; (**d**) fluorescence lifetime of various co-dopant concentration of the sensitizer/emitter; (**e**) the complex picture under single luminescence; (**f**) the separated patterns based on the lifetime components of every pixel; (**g**) the decoded genuine multiplexing information contained in the same overlapping space [59].

The traditional fluorescent color codes are commonly influenced by spectral overlap and background interference, which limits the ability of reading and labeling. In 2013, Jin and coworkers controlled the fluorescence lifetime τ in the microsecond region for further application in independent codes on upconversion nanocrystals (Figure 6a–c) [59]. Ten different nanocrystals with the lifetime range from 25.6 μs to 662.4 μs could be generated in a single-color band, and the new-fabricated strategy was able to precisely decode the individuals with different lifetimes (Figure 6d). The τ pots were very promising to be employed in multiplexing imaging, high-registry cytometry quantification, high-density optical storage, and security identification coding, enlarging the optical multiplexing range by increasing the time dimension of luminescent signs. The new strategy of optical multiplexing and a series of nanolabels and microcarriers were demonstrated, and equipped with individual lifetime labels (Figure 6e–g). Thus, as a new-born potential technology, the novel method can hopefully conquer the challenge of medicine therapy in complicated environments, paving a way for document security and the high-density, long lifetime, and intense stability of data storage.

## 3. Graphene-Based Functional Materials

Graphene is a 2D crystal that resembles as hexagonal network structure with the thickness of a single atomic layer, which is constructed from the tight arrangement of sp^2^ hybrid carbon atoms. Owing to the distinct 2D skeleton, specifical energy band structure, and remarkable carrier transport velocity, the graphene possesses broad prospects in memory storage [60]. In addition, GDs derived from graphene such as graphene oxides (GOs) are equipped with abundant carbonyl, hydroxy, and epoxy moieties on the surface, as well as the numerous conjugated units in the intrinsic frameworks, endowing the GDs with intense fluorescent properties for the fabrication of luminescent devices. In this section, according to the admirable photoelectric performance of GDs, we introduce the GD-based functional materials for the application in the field of optical data storage (Table 2).

**Figure 7:**
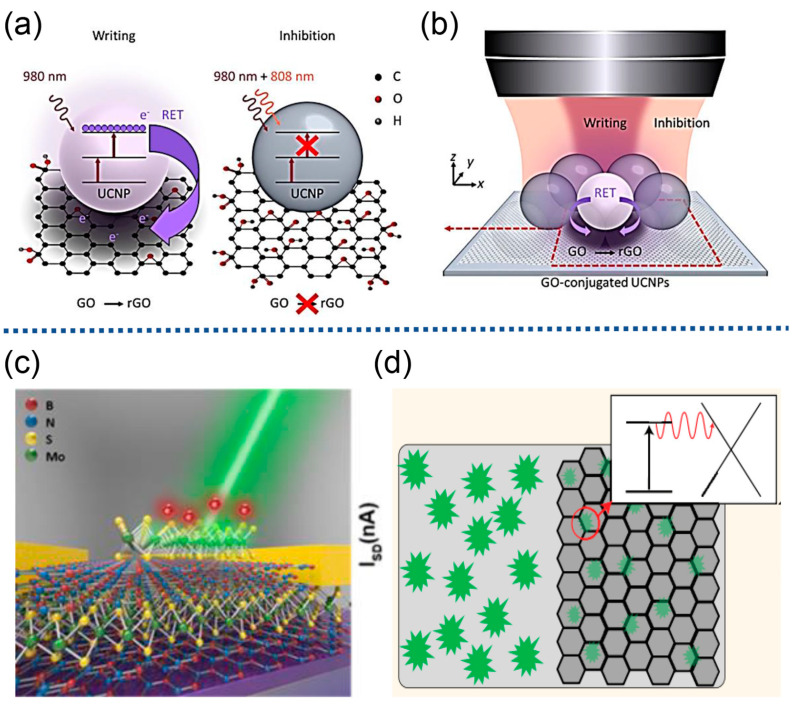
(**a**) The optical writing mechanism of GO-conjugated UCNPs-5 [61]; (**b**) the schematic diagram of the dual-beam super-resolution optical writing; (**c**) the schematic diagram of photoresponsive multilayer MoS_2_ storage [62]; (**d**) the whole optical image of graphene [63].

In 2021, Gu and coworkers used NaYF_4_ mixed with Tm^3+^ and Yb^3+^ to synthesize the UCNPs-5, and deposited the GO material on indium tin oxide (ITO), which were further assembled through the electrostatic interaction by putting the UCNPs-5 solution on the GO material for preparing the GO-contained UCNPs-5 (Figure 7a) [61]. Using GO and UCNPs as substrates, optical writing with a feature size of 50 nm was achieved under dual-beam super-resolution irradiation through the high-energy resonance energy transfer (RET) effect of UCNPs-5, which successfully induced the local reduction of GO flakes. In comparison, the traditional work reported that the nanostructures of fused silica glass required a 10^4^ times higher intensity of beam than the upconversion RET to accomplish the procedure of optical writing. In addition, the fluorescent quenching effect of the reduced GO toward upconversion luminescence was utilized to achieve the high-contrast subdiffraction reading of the recording information (Figure 7b). The reduced GO was capable of performing multiple write/read processes through the oxidation reaction, and the stability of the writing process was enhanced by selecting stable GO and decorating nanocomposites into the recording medium. Furthermore, the UCNPs-5 equipped with high-capacity and low-energy GO conjugation for optical storage are expected to achieve the storage capacity of 700 TB in a single optical disc, opening a new avenue to address the challenge of global data storage.

The optoelectronic memory devices are the new tools that on the basis of optoelectronic signals to operate the on/off state, possessing extensive applications in image sensing and parallel data transmission. Yoo and coworkers reported a MoS_2_-based memory device decorated with the top floating gate of graphene that served as a charge-trapping layer with photoresponsive properties, which was superior to the conventional floating-gate storage devices [62]. The MoS_2_-based memory device with graphene-based top floating gate possessed stable and durable features and was capable of achieving an on/off ratio of 10^6^ by pulsed light irradiation at 405 nm and valve voltage pulses, and the retention time could exceed 10^4^ s (Figure 7c). In addition, the multi-energy optical storage effects derived from the graphene-based top floating gate could be observed under 532 nm or 635 nm pulsed light, suggesting that the performance of the device was related to wavelength and exposure time. The optical storage properties could be explained by the number of electrons transferred from the top floating gate of graphene to MoS_2_. Simultaneously, the barrier height of the electron tunnelling from the floating gate of graphene through the hexagonal boron nitride to the MoS_2_ channel was between 2.33 eV and 3.06 eV, which was favorable for the practical application in optical storage.

In recent years, fluorescence quenching microscopy (FQM) has been used as a new measure for optical imaging of graphene-based flakes in characterization experiments; however, the resolution of FQM remained confined to the diffraction limit of a few hundred nanometers. Stohr and coworkers detected fluorescence in FQM by means of a super-resolution imaging technique, which was similar to STED [63]. The combined technique of fluorescence resonance energy transfer (FRET) and STED, namely FRET + STED, was first proposed for promoting the FQM method, not only displaying ultrahigh resolution, but also allowing the use of visible light. The optical imaging of graphene showed that the improved strategy was capable of achieving a resolution within 30 nm (Figure 7d). Taking advantage of the efficient optical technique based on FQM and super-resolution imaging, the authors adopted the technology to image graphene on Pr:YAG. Notably, the experimental results showed that the optical resolution was below 30 nm, completely satisfying the current requirement of optical imaging of graphene-based thin sheets.

## 4. Diarylethene-Based Functional Materials

Precise writing and nondestructive readouts are indispensable features for advanced optical storage. The diarylethene (DTE)-based fluorescent molecular switches can reversibly change between open ring isomer and closed ring isomer states via alternating UV/visible light irradiation, which is equivalent to photocontrollable molecular-scale digital switches for directing the data recording and reading, having promising prospects in high-density optical data storage by virtue of its excellent thermal stability and fatigue resistance [64]. Generally, the wavelength of reading beam for the DTE will participate in the open/closing ring reaction, exerting severe effects for optical writing and reading. Thus, the functional DTDs combining DTE molecules and nanocomposites are fabricated for pursuing accurate recordings and nondestructive readouts (Table 3).

**Figure 8:**
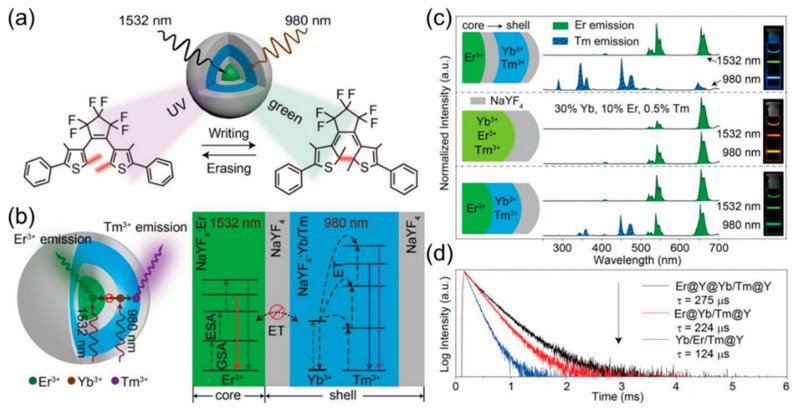
(**a**) Schematic representation optical storage using the core-shell nanoparticle; (**b**) the internal structure of the core-shell nanoparticle (**left**) and the schematic diagram of energy transfer pathways between the lanthanide dopants (**right**); (**c**) upconversion luminescence images of 3 different nanoparticles; (**d**) the luminescence lifetimes of Yb^3+^ doped nanoparticles with different structures [65].

An upconversion nanoparticle NaYF_4_:Er@NaYF_4_@NaYF_4_:Yb/Tm@NaYF_4_ (UCNPs-6) based on core-shell structure was first designed and reported by Liu, Zhang, and coworkers [65]. Through the functionalized modification, the UCNPs-6 decorated with DTE-based luminescent molecule (DTE-1) could be further applied in rewritable optical storage (Figure 8a). The fluorescence spectra showed that the core-shell structure endowed the UCNPs-6 with two emission bands under the excitation of 980 nm and 1532 nm. Notably, the UCNPs-6 exhibited UV light under 980 nm excitation, to direct the cyclization reaction of DTE-1 with the generation of C-C covalent bonds, while the 1532 nm excitation could direct the fracture of C-C covalent bonds with green emission. This stimuli-responsive property provided a convenient method for high-resolution data writing and erasing. The UCNPs-6 was designed by using layer-by-layer assembly to introduce different activators in shells (Figure 8b). The orthogonal upconversion emission was in favor of precisely controlling the optical reaction of DTE-1. In detail, the Er^3+^ was defined in the core to generate green emission under 1532 nm excitation, and the Yb^3+^/Tm^3+^ were co-doped in the shells to produce UV light under 980 nm excitation (Figure 8c,d). Therefore, the results suggested that multi-layer NaYF_4_ was modified by Er^3+^ and Tm^3+^ activators in different layers to achieve upconversion emission under the excitation of 1532 nm and 980 nm, triggering the reversible photochemical reaction of DTE-1. Such capability for selectively pumping Er^3+^ and Tm^3+^ with 1532 nm and 980 nm excitation to generate purified emission paved the way for the rewritable optical storage.

**Figure 9:**
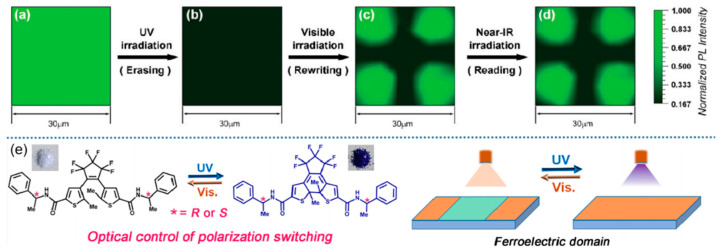
Photo luminescence mapping images of the composite film illustrating (**a**) initial state; (**b**) erasing; (**c**) rewriting; and (**d**) nondestructive readout [66]; (**e**) data storage in diarylethene-ferroelectric crystals [67].

Similarly, Yan and coworkers successfully produced a unique 2D rewritable optical storage medium through the architecture of organic-inorganic hybrid materials [66]. The employment of a DTD-based photochromic modulator enabled the recording medium to be rewritable and durable. Owing to the unique excited property of the as-synthesized β-NaYF_4_:Yb,Er (UCNPs-7), the accessible reading wavelength could be expanded to a near-infrared region. Notably, the DTDs exhibited no observable absorbance under the reading beam, avoiding the problem of damaged information during the readout process. It was noteworthy that the writing, erasing, and reading of the data spots were performed under a single beam of light irradiation on a two-dimensional recording medium, demonstrating the better compatibility of the strategy compared with the contemporary CD/DVD devices (Figure 9a–d). Although this storage system required a multi-wavelength light source, fluorescence emission provided a facile platform with the advantages of low background signal and high sensitivity, which was of great importance to employ super-resolution techniques. In addition, the authors prepared films with different degrees based on monodispersed UCNPs-7 through methods including spin-coating, evaporation-induced self-assembly, and Langmuir–Blodgett techniques. The films prepared by the evaporation-induced self-assembly method had a desirable thickness, roughness, and uniformity, and could be applied in scanning near-field optical microscopy to obtain higher storage density. Thus, controlling the energy transfer and constructing assembled nanoparticles based on such UCNPs can provide a new way for three-dimensional optical storage.

Many traditional studies find it difficult to achieve a contactless integrated process of writing, reading, and erasing in data storage. In order to tackle this problem, Xiong and coworkers synthesized a pair of ferroelectric semiconductor crystals with DTE enantiomers [67]. The as-prepared material could be induced by photoisomerization to drive ring opening/closing reactions and had the ability of light-driven phase transition (Figure 9e). Under visible light, the material was in a ring-opening state, exhibiting white emission. Moreover, upon exposure to UV light, the material transformed into a ring-closing state, emitting blue light. In addition to writing and erasing on the ferroelectric crystals, the material was capable of reading the emitting color to determine the polarization state. Employing the photoisomerization of RR and SS photochromism of the DTE enantiomers, the ferroelectric dominant region could be controllably switched on, illustrating the significant role of reversible photoinduced phase transitions in the optical control of ferroelectric properties. Notably, the bandgap of the as-prepared material could be reversibly modulated between 1.68 eV and 3.26 eV, which was the narrowest bandgap among the reported organic ferroelectrics up to now. Equipped with the excellent tunable properties of adsorption, this work stimulated new thinking for synthesizing the optical-controlled ferroelectric crystals and accelerating the development of photoresponsive ferroelectric devices.

**Figure 10:**
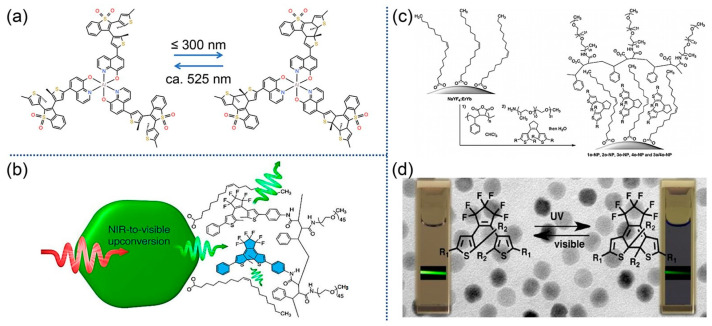
(**a**) The photochromic reaction of Alq_3_ complex [68]; (**b**) the fluorescent quenching mechanism of photoresponsive polymer toward the lanthanide-doped UCNPs [69]; (**c**) the synthetic route to the hybrid nanoparticles based on photoresponsive compounds; (**d**) the excitation wavelength to trigger the ring-closing and ring-opening reaction of DTE [70].

The tris(8-hydroxyquinoline)aluminum (Alq_3_), as a basic moiety for organic light-emitting diodes (OLEDs), has attracted much attention in organic electronics, photoresponsive materials, and organic optical storage materials [71,72,73,74]. Yam and coworkers developed a photoresponsive Alq_3_ complex based on DTE through rational design (Figure 10a) [68]. By incorporating photochromic units with different heterocycles in the Alq_3_ framework and assisting with effective Al(III) complexes for photo cyclization, the photochromic performance and photoswitching efficiency could be easily tuned. This intrinsic photochromic behavior enhanced the electron transport performance and realized photocontrolled electron migration. These complexes could serve as active layers in resistive memory devices. The Alq_3_ complexes containing DTE groups developed in this work possessed good photoinduced cyclization properties. Through changing the heterocyclic bridges on the DTE groups, the color and photochromic properties of the Alq_3_ composites in the closed state could be tuned. Thus, this work introduced photochromic DTEs in the 8-hydroxyquinoline-based organometallic systems, demonstrating the great potential of Alq_3_-based complexes for the fabrication of resistive memory devices.

Subsequently, Branda and coworkers synthesized a DTD-based photoresponsive amphiphilic polymer for encapsulating lanthanide-doped UCNPs, and further prepared a nanoassembly with a strong emission quenching agent (Figure 10b) [69]. The nanoassembly emitted fluorescence in the visible region under 980 nm and switched the conformation of the photoresponsive chromophore attached to the polymer backbone by UV and visible light with reversible modulation. Photon counting experiments showed that the quenching mechanism of the nanoassembly was a combination of FRET and emission–reabsorption effects. Due to the decoration of photoswitching loading, this novel nanoassembly was endowed with higher quenching efficiency. The construction of the nanoassembly will be useful for the design of novel organic–inorganic hybrid nanosystems for the bioimaging and enhancement of a FRET quenching effect in the UCNPs with DTDs. Intriguingly, the amphiphilic polymer shells containing photoresponsive DTE chromophores and UCNPs were prepared by the same group [70]. The water-soluble assembly emitted fluorescence in the visible region under the excitation of 980 nm near-infrared light (Figure 10c). Changing the UV and visible light could control the ring closing/opening reactions of the DTE chromophore to precisely modulate the fluorescence emission (Figure 10d). The polymer shell was able to promote the affinity of the UCNPs with water and preserve a hydrophobic environment conducive to organic photoreactions, and it could serve as an ideal method for preparing light-responsive systems that can be applied in aqueous media, bioimaging, and diagnostics [75].

## 5. Conclusions and Perspective

In summary, the representative works of advanced functional materials related to UCNPs, GDs, and DTDs for optical storage applications have been comprehensively discussed in this review. Meanwhile, the specific applications, advantages, and disadvantages of each system were also elaborated in detail. As mentioned, the three functional systems based on UCNPs, GDs, and DTDs possess great potential for application in optical data storage; however, there are several problems concerning them, so the values of the benchmark parameters, superiorities, and drawbacks are summarized for clear comparison of the three material categories (Table 4).

Although outstanding progress has been made in the field of optical storage in recent years, the three types of advanced functional materials are still at the early stages of research and facing severe challenges, which greatly limit their further development in exploiting novel functional materials for enlarging the capacity of optical storage and manufacturing optical discs for practical use. In order to satisfy the wide requirement of the current digital age, we believe that on the basis of the three categories of functional materials, novel optical storage systems equipped with higher density, larger capacity, longer life, and stronger reliability need in-depth exploration to gain the merits as follows:

(i) Low-cost and high-performance UCNPs: many traditional synthetic routes to UCNPs are not environmentally friendly enough, and the luminescence efficiency of the prepared rare-earth nanomaterials are usually unsatisfactory. The rising AIEgens with strong luminescence can be modified in novel UCNP systems to address the crucial issue [76,77]. Thus, obtaining multifunctional UCNPs with high quantum yields and tunable luminescence is an urgent subject for future research in optical applications [78]. In addition, to realize the industrialized application of UCNPs in practical optical storage, reducing the original cost is a vital part of the subsequent research. Controlling the synthetic time, ambient temperature, environment humidity, and mixture concentration to obtain UCNPs with remarkable uniformity, identical morphologies, and precise periodicity, the low-cost and high-performance UCNPs are expected to be successfully generated.
(ii) Incorporating graphene into other luminescent materials can endow the new composites with lower energy consumption, higher sensitivity, and faster detection speeds. The specific method to prepare GDs with cheap raw materials, controllable structure and properties, and easy steps is short of study, and should be urgently investigated for putting the GD-based optical storage systems into practice. Efficiently growing high-quality graphene for subsequent production is a major issue for GDs to be applied in optical storage devices. It is worth mentioning that exploring the read–write property of optical memory by utilizing the structural transformation between graphene and graphene oxide has been an important research direction in recent years. Notably, the regulation of morphology uniformity is still a major problem in preparing GDs, and subsequent changes related to the reaction temperature, reaction time, and coating method should be carefully considered to address this problem.
(iii) In general, the reported photoresponsive systems based on DTDs require short-wave UV or visible light to activate isomerization reactions of the molecules, which will lead to the crosstalk of information between recording layers during optical writing/reading. In comparison with inorganic composites, the significant downside of organic materials is the less thermal stability and weaker fatigue resistance. Thus, the future design of DTD-based optical storage systems must focus on the DTE materials equipped with intense structure rigidity, strong anti-interference performance, and large conjugated frameworks, which is expected to be achieved by introducing conjugated blocks such as macrocyclic hosts, long chains of polymers, and coordination frameworks, for meeting the requirements of rewritable and non-destructive recordings with large storage capacity, no signal crosstalk, and high signal-to-noise ratio [79,80,81,82].

Particularly, the researchers engaged in optical data storage have also carried on remarkable investigations on the other functional materials such as quantum dots (QDs) [83,84,85,86], glass-based media [87,88], azobenzene derivatives [89,90], and organic dyes [91,92]. Advanced progress has been achieved on the basis of the other functional materials for designing optical memory systems in the laboratory, which is promising to further produce new equipment for replacing the currently applicable storage devices and to be applied in more efficient data storage in the future. For instance, quantum dots have become favorably used for fabricating optical multilevel spin bit devices owing to the combined advantages of good photostability, tunable luminescent features, controlled emission spectrums, large Stokes shifts, and long fluorescence lifetime, which are contributed to optical writing and the architecture of QDs-based optical devices. We strongly believe that these functional materials all exhibit fantastic prospects and they are expected to enhance the recording density and enlarge the storage capacity with more outstanding performance in the near future.

The rapid development of the three types of representative materials provides new ideas for the design of many optical devices, so that the performance of the new-born systems is more diversified and their control mode is more ingenious, and it is believed that the construction and development of new composite materials will have a wider range of application prospects in future optical applications.

## Figures and Tables

**Table 1:** UCNP-based functional systems for optical storage applications.

UCNP-Based Systems	Methods of Preparation	Properties	Ref.
UCNPs-1	Sol–gel pyrolysis technique	Stimulated absorption mechanism and emission in the range of visible light	[54]
UCNPs-2	Solvothermal method	Reducing the requirement of optical depletion for laser intensity	[55]
UCNPs-3	Oxygen-free hydrothermal protocol	Background-free and ultrahigh-sensitivity imaging	[56]
NaYF_4_ nanocrystals	Solvothermal method and crystal growth	Precise control of phase, size, and optical emission features	[57]
Different phases of NaYF_4_:Yb,Er	Thermal decomposition of metal oleate precursors	Defect-reduction strategies of preparing small and brighter UCNPs	[58]
UCNPs-4	Solvothermal method	Realizing the 28 nm super high-resolution	[30]
Lifetime-coded microspheres	Electrostatic interaction	Enlarging the optical multiplexing range by increasing the time dimension	[59]

**Table 2:** GDs-based functional platforms for optical storage applications.

GDs-Based Systems	Methods of Preparation	Properties	Ref.
GO-contained UCNPs-5	Coprecipitation method and electrostatic interaction	Resonance-assisted optical writing and ultrahigh capacity optical storage	[61]
MoS_2_-based memory device	Mechanical exfoliation and polydimethylsiloxane stamping	High on-off ration, long retention time, stable, and durable features	[62]
Graphene-contained Pr:YAG	Electrostatic interaction	Background-free and ultrahigh-sensitivity imaging	[63]

**Table 3:** DTD-based functional materials for optical storage applications.

DTD-Based Systems	Methods of Preparation	Properties	Ref.
UCNPs-6 modified DTE-1	Layer-by-layer epitaxial growth procedure	Highly purified emission and rewritable optical storage	[65]
UCNPs-7 modified DTDs	Electrostatic interaction and self-assembly strategy	Application in scanning near-field optical microscopy for higher storage density	[66]
Ferroelectric crystals with DTE enantiomers	Dropping and spreading the crystal-based precursor on ITO glass substrate to obtain thin film	Achieving a contactless integrated process of writing, reading, and erasing in data storage	[67]
DTE-based Alq_3_ complex	Metal complexation	Photocontrolled electron migration and as active layers in resistive memory devices	[68]
DTD-based photoresponsive amphiphilic polymer	Self-assembly strategy	Combination of FRET and emission reabsorption effects	[69,70]

**Table 4:** The comparison for superiorities and drawbacks of UCNPs, GDs, and DTDs in optical data storage.

Functional Materials	Optical Storage Mechanism	Repeatability	Superiorities	Drawbacks
Upconversion nanoparticles	Spectral hole-burning	Yes	Great thermal stability, good quantum yields, long fluorescence lifetime	Cumbersome preparation and modification
Graphene derivatives	Photoreduction	No	Low cost, high thermal stability, low energy consumption, good sensitivity	Non-uniform morphology and size, inconvenient synthesis
Diarylethene derivatives	Photo-induced ring opening and ring closing changes	Yes	Easy functionalization, convenient reversible reading/writing, great repeatability	Short-wave UV or visible light for activation, moderate thermal stability, weak fatigue resistance

## Data Availability

Data is contained within the article.
